# EVALUATION OF DEMENTIA EDUCATION TRAINING IN GEORGIA NURSING HOMES

**DOI:** 10.1093/geroni/igad104.2392

**Published:** 2023-12-21

**Authors:** Yun-Zih Chen, Jennifer Morgan, Lynnete Okwaro

**Affiliations:** University of Maryland, Baltimore County, Baltimore, Maryland, United States; Georgia State University, Atlanta, Georgia, United States; Georgia State University, Atlanta, Georgia, United States

## Abstract

The increased use and misuse of antipsychotics and psychotropics in nursing homes have been well documented. Person-centered dementia care training that addresses skill development and practice change around communication strategies and non-pharmacologic approaches has the potential to impact the misuse of these drugs as chemical restraints. The goal of this study is to implement and evaluate an online team-based dementia education (Dementia Beyond Drugs: Changing the Culture of Care) program aimed at skill development and practice change. The program was delivered online over six weeks to teams of nursing home staff for a total of 12 hours of synchronous interactive learning. Teams were also assigned application exercises between meetings. Topics included “dementia, dementia beyond drugs, interpersonal approaches, and understanding personal expressions.” This study implemented the training with staff teams from 22 nursing homes in Georgia (N=97). Pre-post data analysis reveals a significant improvement in the nursing home environment and relationships as measured by the Kansas Culture Change Instrument, and increased attention to the use of antipsychotic drugs in staff meetings. This study indicates that team-based online learning has the potential to improve person-centered dementia care practices and attention to the use of non-pharmacological approaches to address behavioral expressions of residents living with dementia. Future research is needed to expand person-centered dementia care education programs to more nursing homes and evaluate their short and long-term effectiveness.

